# Effects of salinization on the occurrence of a long-lived vertebrate in a desert river

**DOI:** 10.1038/s41598-022-20199-3

**Published:** 2022-09-23

**Authors:** Laramie B. Mahan, Lawrence G. Bassett, Adam Duarte, Michael R. J. Forstner, Ivana Mali

**Affiliations:** 1 Department of Biology, Eastern New Mexico University, Portales, NM 88130 USA; 2 Department of Biology, Texas State University, San Marcos, TX 78666 USA; 3 USDA Forest Service, Pacific Northwest Research Station, Olympia, WA 98512 USA; 4 Fisheries, Wildlife, and Conservation Biology Program, North Carolina State University, Raleigh, NC 27695 USA

**Keywords:** Ecological modelling, Freshwater ecology, Conservation biology

## Abstract

The lower Pecos River located in the southwest USA, is a naturally saline river system that has been significantly altered in relatively recent years. Climate change, coupled with anthropogenic disturbances such as dam construction have led to portions of the river becoming more susceptible to increased salinization and declines in water quality. These alterations have been documented to be detrimental to multiple freshwater communities; however, there is a lack of knowledge on how these alterations influence long-lived species in the river, such as freshwater turtles, where the effects can appear over dramatically different temporal scales. The Rio Grande Cooter (*Pseudemys gorzugi*) is a species of concern known to occur in the Pecos River. To understand the current distribution and habitat requirements for *P. gorzugi* in the Pecos River, we used a single-season, single-species occupancy modeling framework to estimate occurrence while accounting for the sampling process. Day of year, water surface area, and water visibility had the greatest influence on the ability to detect the species given a sampling unit is occupied. Conductivity (a measure of salinity) had the greatest influence on the occupancy probability for the species, where sites with higher conductivity coincided with lower occupancy probabilities. This study indicates that increased salinization on the lower Pecos River is a cause for concern regarding freshwater turtle populations within the Chihuahuan Desert.

## Introduction

The Pecos River, USA, is the largest tributary of the Rio Grande River, extending approximately 1500 river km from the southern Sangre de Cristo mountains in northeastern New Mexico and flowing southward through eastern New Mexico and west Texas where it joins the Rio Grande at the Mexico-USA border^1–3^. Geographically, the Pecos River is divided into three sections: the upper-, middle-, and lower-Pecos^1,2^. The hydrological regime varies greatly throughout the river system: (1) the upper Pecos is located within the alpine tundra of northeastern New Mexico, and water is mainly derived from mountain snowmelt; (2) the middle Pecos River stretches from the city of Santa Rosa to the city of Artesia, New Mexico, where water is mainly derived from springs; and (3) the lower Pecos River flows through the Chihuahuan Desert including the Permian Basin, from southeastern New Mexico to southwestern Texas, and southward until it joins the Rio Grande^1^. Water in the lower Pecos River is mainly derived from occasional thunderstorm runoff and it is regulated by several dams and reservoirs constructed in the middle and lower sections of the river^1,2^.

Historically, the Pecos River has been a vital water source to settlers for the irrigation of crops and livestock management, allowing domesticated livestock to survive the arid and otherwise harsh conditions of the Chihuahuan Desert and Permian Basin^3,4^. Native Americans, Spanish explorers, and frontier cattlemen used the river for drinking water for both humans and animals, even though portions were notably salty and foul-tasting^4,5^. The natural salinity of the middle to lower sections of the Pecos River is derived from salts entering the system through dissolved rock deposits (e.g., halite and gypsum) and underground brine^2,4,5^. Prior to development, the river was not too saline for freshwater organisms due to higher flood frequency and stronger streamflow^5^. However, since the late 1800s climate change has, in part, induced a reduction in flood frequency, decrease in streamflow, and increase of evapotranspiration, allowing the persistence of saline groundwater, and subsequently rising salinity levels in the lower Pecos River system^3–5^. Additionally, throughout the Chihuahuan Desert, most years have seen evapotranspiration rates greatly exceeding precipitation rates, resulting in frequent droughts and flash floods when precipitation does occur^4^. The southwestern USA is currently in a megadrought, the cause of which is attributed 19% to anthropogenically induced climate change^6^. Anthropogenic disturbances including oil production, agricultural irrigation practices, and dam construction have further allowed areas of the Pecos River to become more susceptible to excess salt buildup, reduction in historical flows, and an overall decline in water quality (e.g., low flows and accumulation of hazardous chemicals)^5,7^. Dissolved salts within the river system can be stored in lands used for irrigation during low flow events, and subsequently returned highly concentrated through return-flows and in periods of high flow events^1^. Non-native introductions of Saltcedar (*Tamarix* sp.) have proven to be detrimental to Pecos River aquatic habitats, as the trees consume vast quantities of river water and its underlying salts and subsequently deposit the salt back to the water’s surface through leaves^3,4^. From 1890 to 1980, dams and reservoirs were constructed in the Pecos River for flood control and irrigation storage, significantly altering the streamflow: Santa Rosa Dam and Sumner Dam in the middle Pecos, Brantley Dam (took place of McMillan Dam), Avalon Dam, and Red Bluff Dam^2,3^ in the lower Pecos.

The combination of increased salinity, droughts, and diminished flow may have long-term adverse effects on aquatic organisms, and potentially cause a permanent reduction of biodiversity in the Pecos River system^2^. Significant alterations of the Pecos River have negatively altered food webs^8^, fish diversity^5,9,10^, and mussel populations^11^, with some species found to be extirpated or greatly reduced^5,9,10^. However, the consequences of prolonged alterations of the Pecos River have not yet been investigated for long-lived species such as freshwater turtles. Turtles are valuable to their respective ecosystems through involvement in seed dispersal and germination^12^, bioturbation of soils^13^, and biomass contribution^14^. Freshwater turtles serve as bioindicators of environmental quality, as they are known to accumulate chemicals that reside in their respective water systems (e.g., pesticides, polybrominated diphenyl ethers [PBDEs], and polychlorinated biphenyls [PCBs])^15–17^. Despite their importance, turtles are amongst the most threatened groups of vertebrates, and their decline may consequently have detrimental impacts on the ecosystems in which they reside^14^.

**Figure 1:**
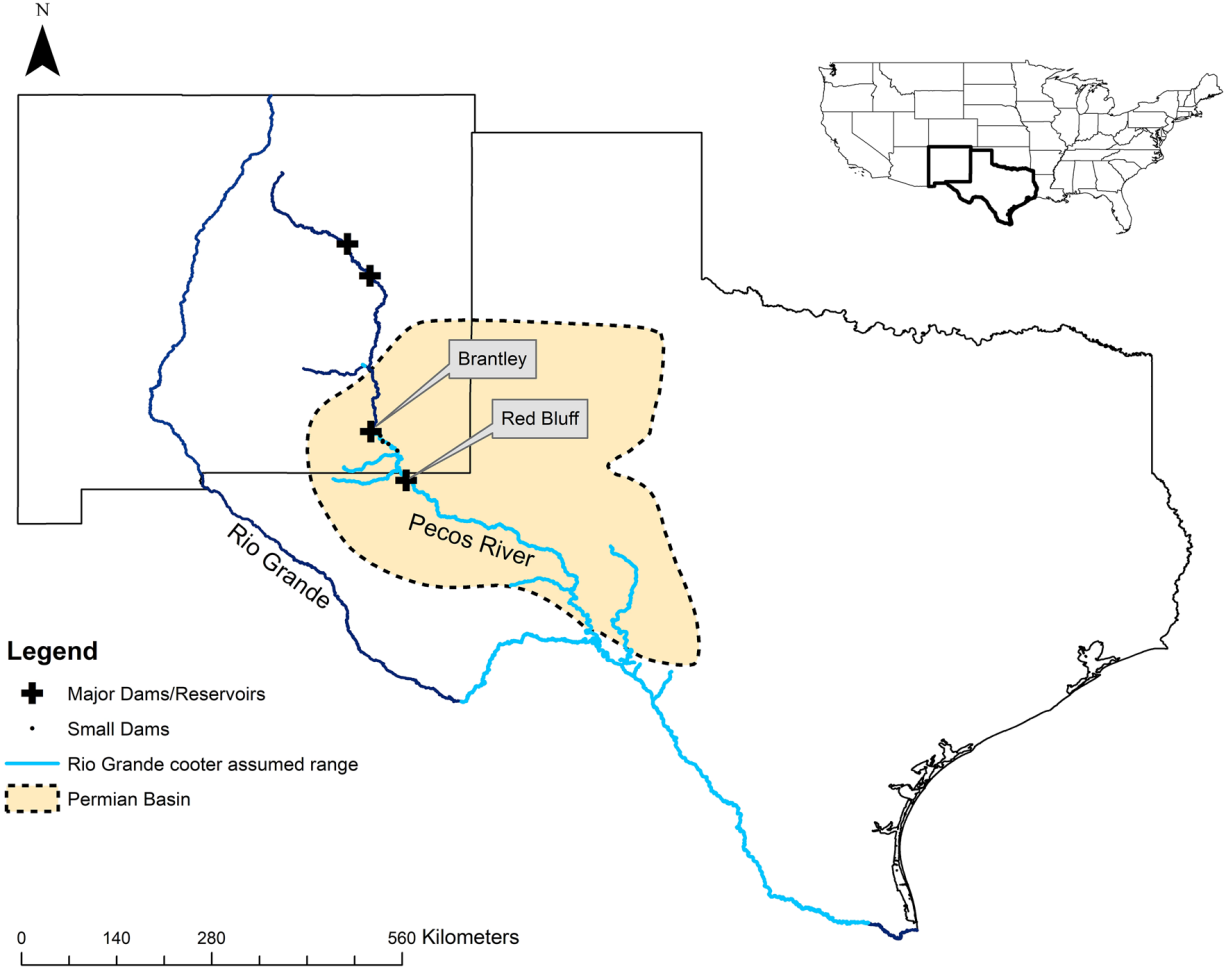
The assumed range of the Rio Grande Cooter (*Pseudemys gorzugi*) along the Rio Grande River watershed and its tributaries in the USA. Major and minor dams/reservoirs are indicated by black plus signs and black dots, respectively. The tributaries of the Rio Grande River in Mexico are excluded.

*Pseudemys gorzugi* is a large riverine turtle belonging to the pond turtle family Emydidae. The species is currently threatened in New Mexico and Mexico and is a species of greatest conservation need in Texas^18–21^. The species was evaluated for potential federal listing under the USA Endangered Species Act^22^, with a final decision in 2022 made to not list the species^23^. Current known threats to *P*. *gorzugi* include habitat degradation through anthropogenic modification of river flow, historical over-exploitation, recreational fishing, and recreational shooting^22,24,25^. As the westernmost species of its genus, *P. gorzugi* is native to southeast New Mexico and southwest Texas, USA, extending to Tamaulipas, Nuevo León and Coahuila in northeastern Mexico^26–28^. In the USA, the species is found along the lower Rio Grande River watershed and its tributaries from the city of Brownsville, Texas to the Big Bend region of west Texas and extending into the Devils and Pecos Rivers (Fig. 1)^27^. In New Mexico, *P. gorzugi* occurs in the lower Pecos River (i.e., downstream of Brantley Dam) including its tributaries, the Black and Delaware River^22,24,26^. Recently, the species was documented 80 km north of Brantley Dam in Berrendo Creek^29^. Primary studies on the species have been conducted in the tributaries of the Pecos and Rio Grande Rivers, especially the Black River in New Mexico and the Devils River in Texas, respectively. The Pecos River represents an important component of the range for *P. gorzugi*, acting as a corridor between the populations in the Pecos River and Rio Grande tributaries throughout New Mexico and Texas. However, the Pecos River itself has never been surveyed across the entirety of the species assumed range^30,31^. A study in New Mexico^30^ reported only four localities where *P. gorzugi* were captured on the main stretch of the Pecos River (with an additional locality at the Black-Pecos River confluence) while a study in Texas^31^ found the species at both of their surveyed sites. Neither of these studies, however, assessed relationships between habitat characteristics or environmental variables and the occurrence of the species while accounting for imperfect detection (i.e., failing to detect a species, given it is present).

As the Pecos River has been subjected to heavy anthropogenic modification in recent years, it is important to assess the occurrence of long-lived organisms such as *P. gorzugi* that may lack the ability to withstand increases in adult mortality^32^. Chelonians are especially vulnerable to sources of additive mortality due to their delayed sexual maturity and low annual recruitment^32–34^. Though *P. gorzugi* may appear abundant in the tributaries of the Pecos and Rio Grande Rivers, estimating the current distribution of the species along the Pecos River itself provides insight to potential changes from historical distribution, and environmental factors that influence the presence or absence of the species. We sought to conduct large-scale monitoring across the entirety of the potential distribution of *P. gorzugi* in the Pecos River and estimate the species’ occurrence by using a single-season, single-species occupancy modeling framework. We aimed to determine which environmental characteristics most influence the occurrence of *P. gorzugi* and use the results to better understand habitat requirements and aid in the potential conservation for the species.

## Results

**Figure 2:**
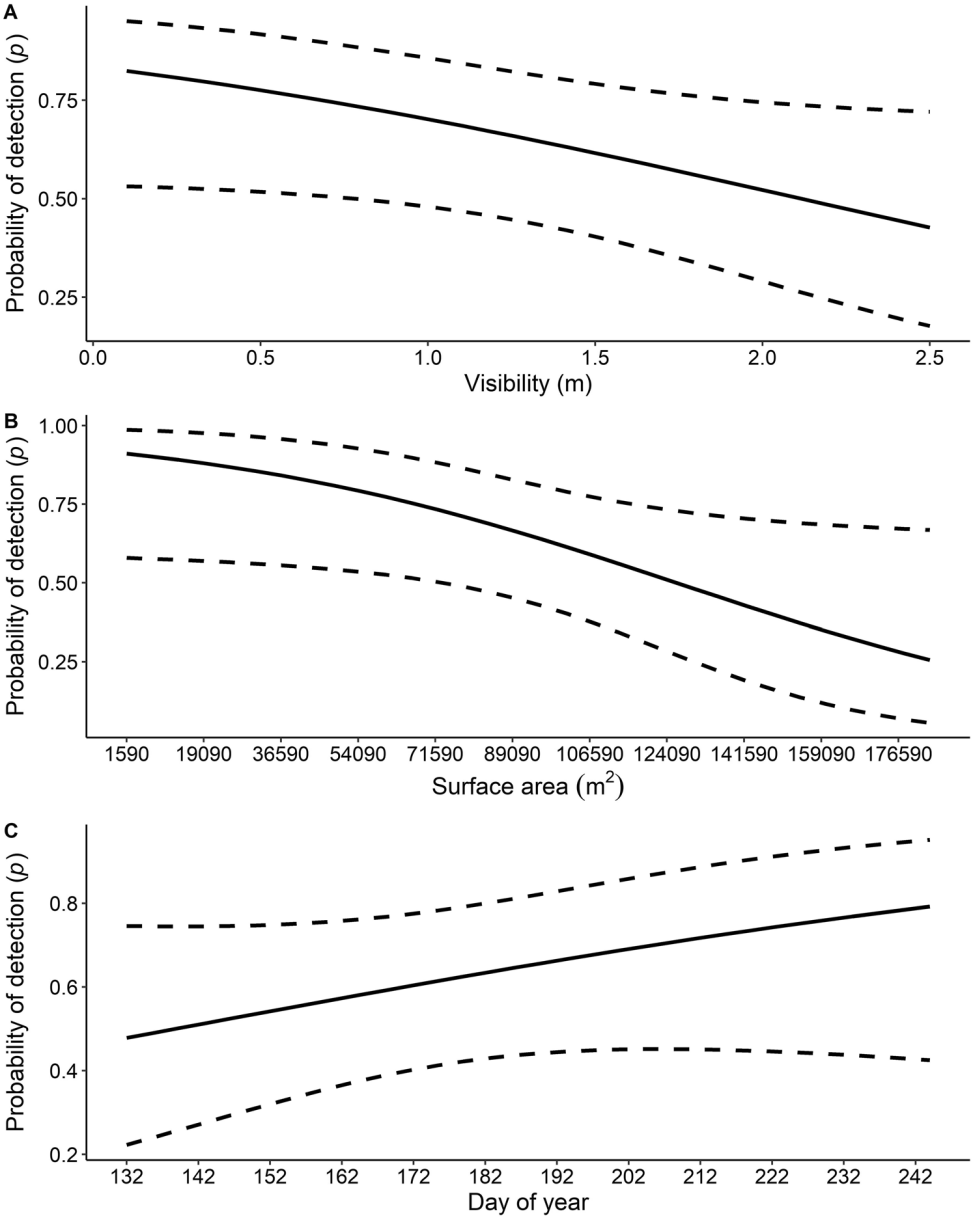
The probability of detecting Rio Grande Cooter (*Pseudemys gorzugi*) along the lower Pecos River, USA given that they were present, calculated from the best-fit model based on survey data from the summer months (May–August) 2020 and 2021.

**Figure 3:**
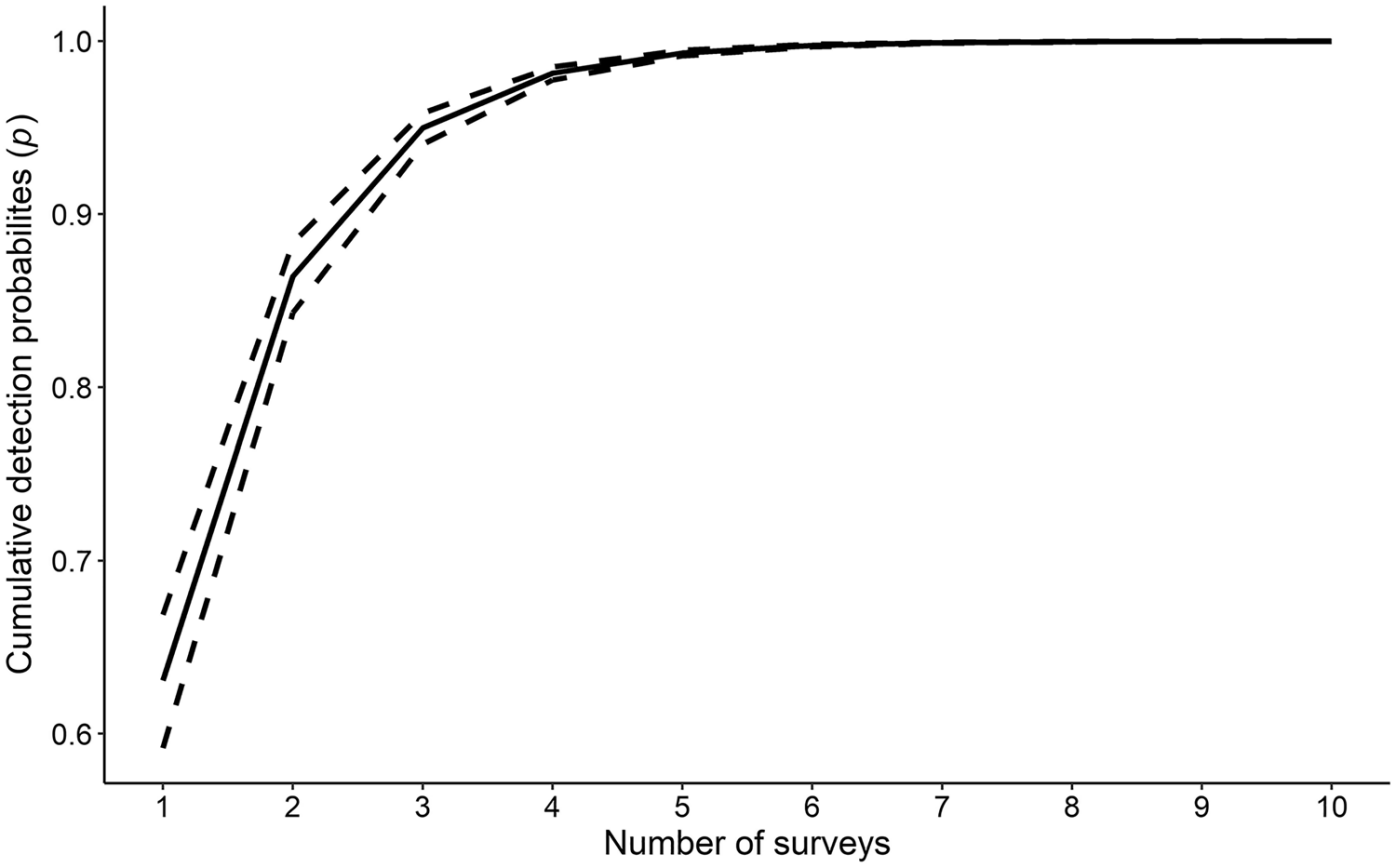
The cumulative detection probability of Rio Grande Cooter (*Pseudemys gorzugi*) along the lower Pecos River, USA, based on covariates (e.g., visibility, day of year, surface area) from the best-fit model using survey data from the summer months (May–August) 2020 and 2021. Note that 45 hoop-net traps were deployed for each survey occasion.

During 2 years of sampling (2020–2021), 32 sites were surveyed over three survey occasions each, for a total of 96 site visits. We captured 60 unique *P. gorzugi* from 14 of the 32 sampling sites. The estimated proportion of occupied sites was 48.08% (95% ci = 43.75–59.38%). Overall, male:female sex ratio was 2:1 and only two turtles were considered juveniles (< 110 mm straight line carapace length)^35^. The best-fit model (Table 1) indicated that the detection probability was most influenced by water visibility, water surface area of a survey site, and day of year. The odds of detecting *P. gorzugi* given a sampling unit was occupied decreased by 1.75 for every 0.41 m increase in visibility (Fig. 2a), and 2.79 for every 31,768 m^2^ increase in water surface area (Fig. 2b). In addition, the odds of detecting *P. gorzugi* given a site was occupied increased by a factor of 1.54 for every 30 d increase in day of year (Fig. 2c). Additionally, according to the third-ranked model (Table 2), the odds of detection increased by a factor of 1.16 for every 3.33 °C increase in water temperature. Using our survey protocol, the cumulative detection probability for *P. gorzugi* ranged from 0.63 (95% ci = 0.59–0.67) to 0.86 (95% ci = 0.84–0.88) after one to two surveys, respectively (Fig. 3). Detection probabilities reached 0.95 (95% ci = 0.94–0.96) to 0.99 (95% ci = 0.99–0.99) after three to five surveys, respectively (Fig. 3).

**Table 1:** The best fit model estimating occupancy and detection probabilities of Rio Grande Cooter (*Pseudemys gorzugi*) in the lower Pecos River with parameter estimates, standard errors (se), and 95% confidence intervals (ci) reported on the logit scale.

Parameter	Estimates (se)	95% ci	
		Lower	Upper
**Detection (*p*)**			
Intercept	0.626 (0.443)	− 0.24	1.49
Day of year	0.431 (0.348)	− 0.25	1.11
Water surface area	− 1.026 (0.518)	− 2.04	− 0.01
Visibility	− 0.557 (0.313)	− 1.17	0.06
**Occupancy (ψ)**			
Intercept	− 1.72 (1.13)	− 3.94	0.49
Conductivity	− 4.98 (2.44)	− 9.77	− 0.20

**Table 2:** Final model set based on a model selection process that used Akaike Information Criterion corrected for small sample size (*AICc*) to test the probabilities of detection (*p*) and occupancy (*ψ*) of Rio Grande Cooters (*Pseudemys gorzugi*) in the lower Pecos River, USA.

Predictor	*K*	AICc	Δ AIC	AIC Wt
*p* (day of year + water surface area + visibility) *ψ* (conductivity)	6	82.03	0.00	0.201
*p* (visibility) *ψ* (conductivity)	4	83.45	1.42	0.099
*p* (water surface area + water temperature + visibility) *ψ* (conductivity)	6	83.47	1.44	0.098
*p *(.) *ψ* (conductivity)	3	83.50	1.46	0.097
*p* (atmospheric conditions + day of year + water surface area + visibility) *ψ* (conductivity)	7	85.28	3.25	0.040
*p* (day of year + water surface area + visibility) *ψ* (basking structures + conductivity)	7	85.31	3.28	0.039
*p* (day of year + visibility) *ψ* (conductivity)	5	85.45	3.42	0.036
*p* (day of year) *ψ* (conductivity)	4	85.54	3.51	0.035
*p* (water temperature) *ψ* (conductivity)	4	85.79	3.75	0.031
*p* (atmospheric conditions + visibility) *ψ* (conductivity)	5	85.90	3.86	0.029
*p*(.) *ψ* (conductivity + depth)	4	86.05	4.02	0.027
*p* (atmospheric conditions) *ψ* (conductivity)	4	86.07	4.04	0.027
*p* (presence of other turtles) *ψ* (conductivity)	4	86.08	4.05	0.027
*p*(.) *ψ* (conductivity + flow)	4	86.10	4.07	0.026
*p*(.) *ψ* (basking structures + conductivity)	4	86.12	4.08	0.026
*p* (water temperature + visibility) *ψ* (conductivity)	5	86.18	4.15	0.025
*p* (presence of other turtles + visibility) *ψ* (conductivity)	5	86.19	4.15	0.025
*p* (visibility) *ψ* (conductivity + depth)	5	86.22	4.18	0.025
*p* (visibility) *ψ* (conductivity + flow)	5	86.24	4.20	0.025
*p* (visibility) *ψ* (basking structures + conductivity)	5	86.28	4.24	0.024
*p* (water surface area + water temperature + visibility) *ψ* (basking structures + conductivity)	7	86.75	4.71	0.019
*p* (water surface area + water temperature + visibility) *ψ* (conductivity + flow)	7	86.77	4.74	0.019

**Figure 4:**
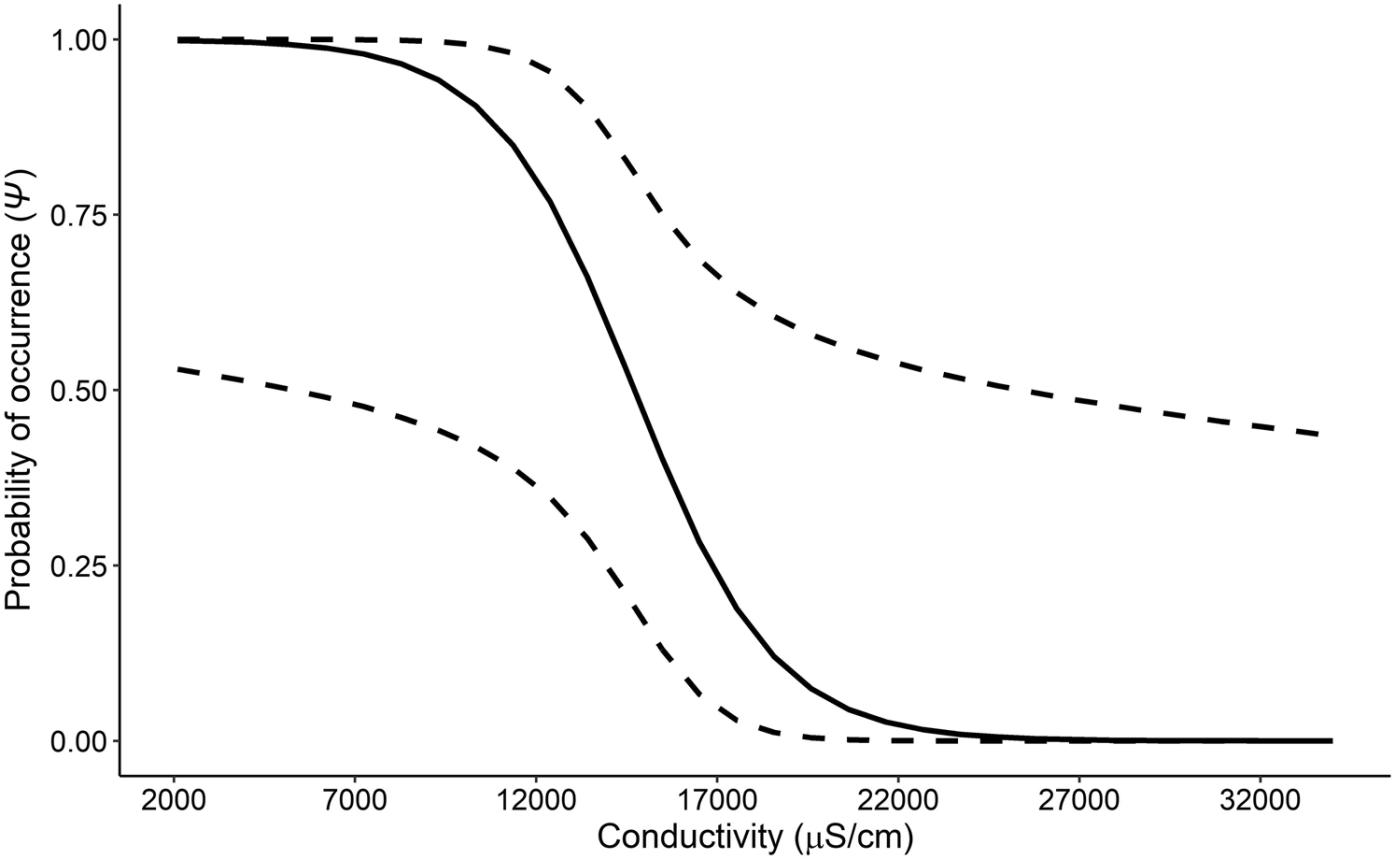
The probability of Rio Grande Cooter (*Pseudemys gorzugi*) occurrence along the lower Pecos River, USA, calculated from the best-fit model based on the survey data from the summer months (May–August) 2020 and 2021. The 95% confidence intervals are indicated by dashed lines.

**Figure 5:**
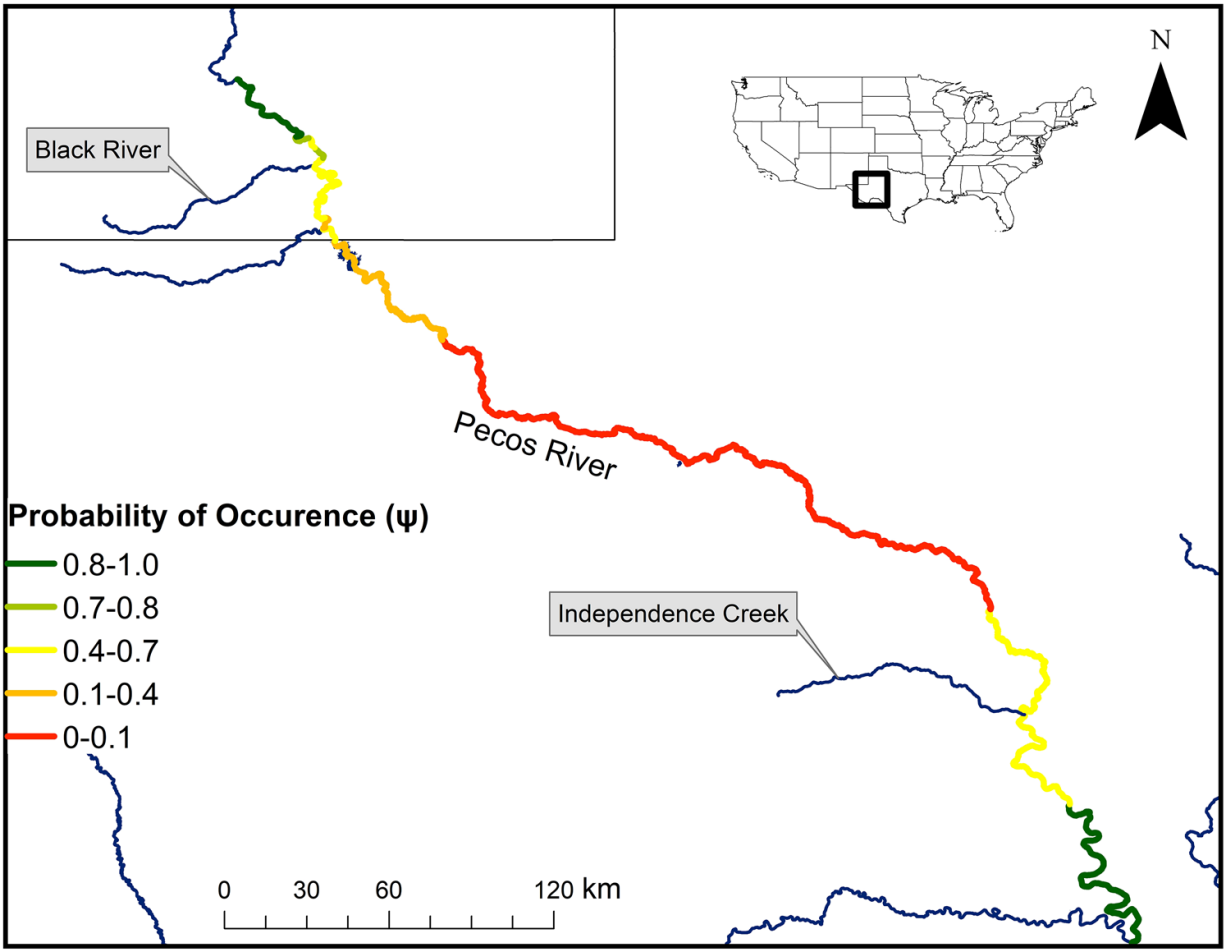
The probability of occurrence for Rio Grande Cooter (*Pseudemys gorzugi*) along the lower Pecos River, USA. Mean occupancy probabilities were calculated between each survey site to visualize potential occurrence probabilities between each survey stretch.

The probability a site was occupied by *P. gorzugi* was most influenced by conductivity (Table 1), with the odds a sampling unit being occupied decreased by a factor of 146 for every 8719 µS/cm increase in conductivity (Fig. 4). Of all recorded water parameters, conductivity varied the most, with a range of 1424–37,397 µS/cm. Conductivity levels gradually increased from the upstream-most sites to downstream-most sites, with relatively sharp spikes downstream from a dam. Conductivity levels decreased where the Pecos River reached confluences with rivers and creeks. Estimated probabilities of occurrence for *P. gorzugi* were the highest at sites above the Black River confluence in New Mexico, and near the Independence Creek and Rio Grande confluences in Texas, where conductivity levels were generally lower (Fig. 5).

## Discussion

This study assesses the distribution of a desert freshwater turtle in one of the most anthropogenically altered river systems of the American southwest. This is the most extensive study on *P. gorzugi* on the lower Pecos River, and the only one to do so while accounting for the sampling process. *Pseudemys gorzugi* is of particular interest given its relatively small distribution and imperiled conservation status in comparison to other species found in this river system. The decision to not list *P. gorzugi* was based on assessments from detection-only data^23^, though the current detection/non-detection data along with model-based estimates of the species occurrence along the Pecos River itself would have been beneficial in evaluating the conservation status of the species along a major portion of its range. *Pseudemys gorzugi* is less tolerant of brackish waters, as lower occupancy probabilities were associated with higher conductivity levels (> 10,000 µS/cm). A distributional gap for *P. gorzugi* in the Pecos River has been speculated for many years but the extensive study design herein has revealed that higher conductivity levels may contribute to the now documented distributional hiatus of at least ~ 390 km of the Pecos River from Loving and Reeves Counties to Crockett County, Texas, USA (portions of the river with the lowest occupancy probabilities; Fig. 5). Crucially, this area may act as a connectivity barrier to the once continuous population of this turtle from New Mexico into Texas. A population genetic study found that New Mexico and Texas populations of *P. gorzugi* are relatively homogenous, indicating that the distribution was once continuous^36^. Therefore, we suggest that the distributional gap found in this study is anthropogenic, and that the populations may no longer be connected, which has implications for the resiliency of *P. gorzugi* along the Pecos River. Additionally, this may imply isolation of the Black River population from the rest of the species range. This suspected gap, along with rapid changes in environmental conditions and additive anthropogenic barriers have potential to influence the representation of *P. gorzugi* in the Pecos River system.

While *P. gorzugi* was detected at 43.75% of the sites, the estimated proportion of sites occupied is only slightly higher, at 48.08% (95% ci = 43.75–59.38%). Day of year, surface area, and water visibility at the time of survey most influenced detection probabilities, which provides insight to survey efficiency for the species. Detection probabilities were higher later in the trapping season around July and August (Fig. 2c). *Pseudemys gorzugi* was more likely to be detected if water visibility was low, suggesting that the turtles are more likely to enter the traps if they cannot visualize them in the water. Probabilities for detection were higher with lower water surface area, a proxy for trap density, which indicates that useful trapping methods for the species on the Pecos River could include high density hoop-net trap surveys^37^. Although the correlation between surface area and conductivity for each survey occasion was not strong (|*r*|< 0.7), some sites with lower conductivity levels had both high and low surface area, and sites with higher conductivity had lower surface area surveyed. With our survey design (45 traps for two consecutive days per survey occasion), cumulative detection probabilities were relatively high, with a probability of detection of 0.63 after only one survey (Fig. 3).

River salinization can contribute to physiological stress and mortality of organisms by altering the osmotic balance in tissues and cells^8^. Historical values of conductivity (proxy for salinity) on the lower Pecos River are not known. However, in the late 1990s and early 2000s, a study recorded conductivity values in select portions of the river from Red Bluff Dam to Girvin, Texas, as a part of the Pecos River Ecosystem Monitoring Project^38^. Recorded values in these portions of the river overlap seven of our sampling sites. By comparing mean conductivity levels recorded from 1999 to 2005^38^, it is evident that the measurements taken more than two decades ago are lower than the values reported herein (~ 194–16,000 μS/cm increase). For example, mean conductivity levels increased from ~ 9900 to ~ 10,094 μS/cm near Red Bluff Dam and from ~ 8000 to ~ 20,467 μS/cm approximately 152 river km downstream from Red Bluff Dam. Freshwater organisms are typically not adapted to such high salinity levels, and high concentrations of salts can be toxic^8,39^. Studies have shown that excess salinity can alter food webs in freshwater systems^8,40^, and can trigger trophic cascades in ecological communities^40^. Specifically, declines in zooplankton and increases in phytoplankton have been reported in systems with excess salinity and the presence of fish^40^. In systems with high salinity and the absence of fish, decreases in filamentous algae were reported^40^. In the lower Pecos River, the food web composition varies greatly^8^. Trophic diversity is highest near the spring fed tributaries, where the conductivity is lowest^8^, which is concurrent with higher occupancy probabilities for *P. gorzugi* reported herein. Throughout the main stem of the river, however, terrestrial organic matter input is low and there is a lower functional diversity, with the predominant species being salt-tolerant benthic macroinvertebrates and non-native euryhaline fishes^8^. *Pseudemys gorzugi* diet consists of algae, dicot vegetation, and arthropods^41–43^. Therefore, in addition to the apparent low tolerance of *P. gorzugi* to high salinity levels, it is also plausible that salinity along with the reduction in trophic diversity and potential lack of food source items has collectively contributed to the absence of the species in the main stem of the river (i.e., where occupancy probabilities are lowest). Worth noting is that no turtles of any species were captured at the five sites with the highest conductivity levels (19,500–37,397 µS/cm), indicating negative impacts on even generalist turtle species, such as *T. s. elegans* and *A. s. emoryi*. Furthermore, at one of the five sites, salt crystal formations were present on the vascular plants that grew along the riverbank (i.e., Saltgrass [*Distichlis stricta*]). There were also three sites where crude oil was present on the vegetation (e.g., Saltcedar [*Tamarix* sp.]) along the riverbank and on the surface of the water. This provides evidence that pollution and high salinity in the Pecos River may have negative implications on the entire freshwater turtle community, which warrants further investigation.

Dams can have profound effects on freshwater biodiversity; however, these effects have not been extensively studied in freshwater turtle populations, especially threatened species. A recent literature review^44^ on the effects of dams in turtle populations found only 43 published studies and emphasized the need for more evaluations for these long-lived organisms. Most of these studies focused on flow changes, barrier effects, and nesting success influenced by dams^44^. Previous reports on the lower Pecos River indicate climate change and anthropogenic processes including dam construction have significantly altered stream flow in relatively recent years^2,3,5^, subsequently increasing salinity levels throughout the river. Our study is a significant contribution to this important conservation topic, as it is the first to demonstrate how these riverine changes can in turn affect the distribution of a near threatened freshwater turtle. Turtle conservation in the Pecos River system would benefit from future studies focused on heavy metal contamination of turtles and an evaluation of whether the apparent gap in distribution found in this study has any consequences on species genetic diversity.

## Methods

This study was conducted on the lower Pecos River in New Mexico and Texas, USA, between 2020 and 2021. A total of 32 sites were surveyed. Upstream of Red Bluff Dam (Texas-New Mexico border), 17 sites were selected based on available public land access and lands managed by the Bureau of Land Management (BLM). Distances between each site ranged from ~ 3 to 26 river km. Downstream of Red Bluff Dam, site selection was limited to bridge crossings (*n* = 13) and river access permission granted by the National Park Service (*n* = 2) and a private landowner (*n* = 1) as 95% of Texas is privately owned. Distances between downstream sites ranged from ~ 4 to 121 river km. Each site was sampled for three survey occasions within a season.

Reliable methods to capture *P. gorzugi* involves high intensity surveys using baited hoop-net traps^37^. To capture turtles, we deployed 45 standard hoop-net traps (50.8 cm diameter and 2.54 cm mesh size; Memphis Net & Twine Co. Memphis, Tennessee, USA), baited with sardines, for 48 h per site, per survey occasion. Additionally, to conserve and evenly distribute resources, every third trap contained a single leaf of romaine lettuce. Traps were checked within the first 24 h, and subsequently pulled at 48 h. Trap theft occurred at two sites, and personnel limitations at another site resulted in the traps remaining in the water for an additional 24 h. The overall trap effort included a range of 269–315 trap days per site.

Environmental and habitat conditions that were hypothesized to influence the occurrence and detection of *P. gorzugi* were recorded (Table 3). Water quality parameters measured at each site included pH, conductivity (µS/cm), nitrates (mg/L), dissolved oxygen (mg/L), flow (m/s), and temperature (°C). Conductivity, pH, nitrates, and dissolved oxygen were measured using YSI Pro Plus Multiparameter instruments (YSI Incorporated, Yellow Springs, OH, USA). Conductivity is used as a proxy for salinity, as conductance refers to the water’s ability to create an electrical current through ions (i.e., dissolved salt ions)^45^. Salinity in the lower Pecos River is mainly attributed to NaCl ions derived from underground brine and halite crystal formations^2^. Conductivity and pH were recorded once per site, per survey occasion. Due to probe availability and limited access to the previous year’s sites (e.g., flood damage to dirt roads in 2021), nitrates and dissolved oxygen were measured only in 2021. To accommodate for missing nitrate and dissolved oxygen measurements for the 2020 sites, values were estimated using the measurements of the nearest 2021 site (closest and furthest distances between the sites were ~ 3 river km and ~ 94 river km, respectively), or the mean values of two 2021 sites of similar distance located on either side of the 2020 site (< 1 river km difference in distances).

**Table 3:** Mean, standard deviation (in parentheses), and range for continuous covariates and frequency for categorical covariates used in a single-season, single-species occupancy model to estimate detection (*p*) and occupancy (ψ) probabilities for Rio Grande Cooter (*Pseudemys gorzugi*) in the lower Pecos River, USA.

Covariate	Parameter	Type	Summary
Flow	*ψ*	Continuous	Mean (SD): 0.11 m/s (0.17 m/s) Range: 0–1.1 m/s
Width	*ψ*	Continuous	Mean (SD): 51.66 m (53.23 m) Range: 3–346 m
Depth	*ψ*	Continuous	Mean (SD): 1.38 m (1.15 m) Range: 0.1–6 m
Conductivity	*ψ*	Continuous	Mean (SD): 10,889.02 µS/cm (8719.1 µS/cm) Range: 1424–37,397 µS/cm
Dissolved oxygen	*ψ*	Continuous	Mean (SD): 78.1 mg/L (25.4 mg/L) Range: 21.1–156 mg/L
Landscape condition	*ψ*	Continuous	Mean (SD): 0.56 (0.24) Range: 0.12–0.95
Basking structures	*ψ*	Categorical	27 present 5 absent
Aquatic vegetation	*ψ*	Categorical	14 present 18 absent
Water visibility	*ψ*, *p*	Continuous	Mean (SD): 0.56 m (0.5 m) Range: 0–3.5 m
Water surface area	*p*	Continuous	Mean (SD): 29,122.78 m^2^ (31,767.47 m^2^) Range: 1598–183,727 m^2^
Water temperature	*p*	Continuous	Mean (SD): 27.41 C (3.26 C) Range: 19.65–34.45 C
Day of year	*p*	Continuous	Mean (SD): 184.49 d (20.28 d) Range: 132–244 d
Atmospheric conditions	*p*	Categorical	73 sunny days 23 clouded or rainy days
Visual presence of other turtles	*p*	Categorical	51 present 45 absent

Flow was recorded three times per site, per survey occasion. For survey sites in New Mexico, flow was measured by inserting a Flowatch® Flowmeter (JDC Instruments, Switzerland) into the river and recording the maximum flow after 15 s. Flow in Texas was measured by inserting an OTT MF pro meter (OTT HydroMet, Germany) and recording the minimum and maximum flow for 15 s. Water temperature was recorded every 4 h by attaching a HOBO Pendant^®^ MX Temperature/Light Data Logger (ONSET, Bourne, MA, USA) to the third hoop of the hoop-net trap. There were malfunctions of HOBO loggers at six sites, during one survey occasion each. For the sites and survey occasions where temperature recordings failed, linear regression equations were created using recorded water temperatures of the other survey occasions at each site of interest and the corresponding air temperatures from the nearest approximate time and location of survey^46^. Air temperatures were accessed from timeanddate.com, which includes hourly weather recordings (temperature, precipitation, pressure, cloud cover etc.) of the nearest city derived from forecasting models by CustomWeather©. Additionally, measurements are retrieved by CustomWeather© from stations run by the Meteorological Assimilation Data Ingest System (MADIS) and the World Meteorological Association (WMO). Missing values were then estimated using the linear equations. Additionally, we documented the daily atmospheric conditions of each site, per survey (cloudy or rain or sunny and partly cloudy).

The species is thought to prefer deep, slow moving pools^19^, therefore depth was measured three times per site, per survey occasion approximately in the center of the river at the beginning, middle, and end of the surveyed stretch by submerging a 10-pound dumbbell weight with a nylon rope marked every 0.5 m. Surface area (m^2^), a proxy for trap density given the consistency in traps deployed each survey occasion, was calculated per site, per survey occasion using the polygon tool in Google Earth Pro. Polygons were created by outlining the length of each surveyed section of the river using Google Earth Pro satellite imagery (2016–2019) in conjunction with the GPS coordinates of the first, middle, and last traps as reference points. To measure river width (m), 15 evenly spaced lines were created within each polygon and the minimum, maximum, and mean measurements were documented once per survey occasion. Water visibility (i.e., turbidity) was measured three times per site, per survey occasion to the nearest 0.25 m by submerging a Secchi disk and recording the depth at which visibility was lost and regained.

As *P. gorzugi* rely on basking for thermoregulation year-round^47^, the presence/absence of basking structures (i.e., fallen logs) was recorded categorically. *Pseudemys goruzgi* is also thought to prefer areas abundant with aquatic vegetation for cover, foraging, and basking^26,27^. Therefore, vegetation was documented categorically based on the presence or absence of emergent aquatic vegetation at each site (cattails [*Typha* sp.] and giant reed [*Arundo donax*]). We recorded the visual observation of any turtle species (i.e., turtle heads above water) categorically, as a proxy for turtle activity (e.g., foraging activity). Although *P. gorzugi* are known to exit the water for nesting, there are no specific studies on nest distances from water for the species. A preliminary study^48^ reported nest distances of approximately 2–36 m from water, indicating that surrounding landscape use may be of importance. To account for surrounding landscape condition of each site, we used the NatureServe Landscape Condition Model (LCM). The LCM uses spatial data from LANDFIRE, USGS ReGap, and National Land Cover Data in the USA to rank the landscape according to the ecological conditions (from 0 [poor] to 1 [excellent]) as a metric of habitat integrity^49^. Values were calculated for 100 m buffers around each site (i.e., water surface area polygon) created in ArcMap^50^.

Occupancy models are a statistical approach used to estimate relationships between ecosystem conditions and the occurrence of species while accounting for imperfect detection^51,52^. Occupancy modeling uses coupled Bernoulli processes and replicate detection/non-detection survey data to estimate occupancy (*ψ*) and detection (*p*) probabilities of a species, respectively^53^. Occupancy probability refers to the probability that a sample unit within the larger study area selected at random is occupied by a species of interest, and detection probability refers to the probability of detecting the species on a survey occasion, given that the sample unit is occupied by the species of interest^53,54^. The probabilities are explained through explanatory variables using logistic regression^53^. This sampling design was created to meet the assumptions of occupancy models^53^: (1) the occupancy state does not change within a single-season (May–August) at a sampling unit; (2) detection probability was independent across sites and survey occasions, using a minimum of 3 km distance between sites and 27 days between survey occasions; (3) the detection and occupancy probabilities were explained through the site-specific and survey-specific covariates chosen based on the study objectives and natural history of the species (Table 3); and (4) false positives do not occur due to proper species identification. Traditionally, *P. gorzugi* was considered a sedentary species making short distance movements (~ 300 m) within their habitat^22,26^. More recently, movements of ~ 1.2 km were reported over short-term periods^55^, while long-distance movements can also occur over longer time periods^56^. We used over double the maximum recorded short-term distance moved as our minimum distance between sites to maximize our ability to meet the closure and independence assumptions of occupancy models.

We fit single-season, single-species occupancy models using detection/non-detection *P. gorzugi* data collected from the 32 sites using the *unmarked* package in *program R*^57,58^ to estimate detection and occupancy probabilities. As turtles are long-lived, we used site-specific covariates assuming they are representative of long-term habitat conditions (Table 3): mean pH, mean conductivity, nitrates, dissolved oxygen, mean water visibility, maximum flow, maximum depth, vegetation type (0 = no aquatic vegetation, 1 = aquatic vegetation), the presence/absence of basking structures, and the mean landscape condition value. Survey-specific covariates that could vary across survey occasions included the day of year (ordinal date), observable atmospheric conditions, visual presence/absence of turtles, water temperature, water surface area, and water visibility at time of survey (Table 3). We compared the mean water temperatures of each site and found the coefficient of variation to be 0.06. Therefore, we felt that there was too little variation in temperatures at the site level to include as a site-specific covariate.

Prior to fitting models, all continuous covariates were standardized to have a mean of zero and a standard deviation of one for comparison across a common scale. Covariates were then checked for collinearity using Pearson’s correlation coefficient. Nitrates and pH were excluded from the model as they were highly correlated with other parameters of interest (|*r*| > 0.7). Models were then constructed using a sequential-by-submodel strategy^59^. First, we compared all possible models for detection while holding occupancy constant. The detection models were compared using Akaike Information Criterion values corrected for a small sample size (AICc)^60^, where we considered submodels important if they had a ΔAICc < 5. We then carried important detection probability submodels forward and fit them with all possible submodel combinations for occupancy, while removing any combination of covariates that resulted in convergence issues. Our final model set was limited to the submodels that had a ΔAICc < 5 in each step of this process, however we only consider the models with ΔAICc < 2 as our top ranked models with similar support (i.e., competing models). This approach was used because it has been shown to recover the top-ranked model, recover a substantial portion of the total AICc model weight, and reduce the number of models fit when compared to fitting every combination of covariates for a simple occupancy analyses^59^. Odds ratio was used to interpret the effects of the top covariates, as effect sizes remain constant across changes in the predictor variables (i.e., covariates)^61^. Using the best-fit model, we examined the predicted occupancy probabilities at each survey site. We calculated the mean occupancy probability of two sites (i.e., site 1 vs. site 2, and site 2 vs. site 3, etc.) to approximately visualize occupancy probabilities between each survey stretch (Fig. 5).

With the best-fit model we also estimated the number of surveys needed to achieve different levels of cumulative detection probability (p*), given the species was present at a survey site (Fig. 3). In particular, we used the best fit detection probability model to estimate the detection probability (mean and variance) for each survey occasion in our study. Using the package *mvmeta*^62^ in *program R*^58^, a method-of-moments estimator for multivariate random-effects meta-analysis^63^ was used to combine these survey occasion specific estimates and calculate an overall mean (and variance) detection probability per survey occasion. The cumulative detection probability was then calculated as p* = *1 – (1 – p)*^*n*^, where *n* is the number of surveys. We incorporated uncertainty in our estimates of p* using a parametric bootstrap simulation approach where we randomly sampled *p* from a beta distribution using the overall mean and variance estimates for detection probability. Scenarios were run for a number of surveys ranging from one to ten, in increments of one. Combinations were then run for 10,000 iterations, and subsequently summarized by their means and 0.025th and 0.975th percentile (i.e., a 95% confidence interval).

### Ethics declarations

Research was approved with permits from the New Mexico Department of Game and Fish (3621), Texas Parks and Wildlife Department (SPR-0615-085/SPR-0102-191), and National Park Service (AMIS-2021-SCI-0005), as well as Eastern New Mexico University and Texas State University IACUC (2019-0226-01A1 and 2015-004, respectively). All procedures were performed in accordance with the relevant guidelines and regulations of each institution. ARRIVE guidelines: not applicable.

## Data Availability

The datasets generated and/or analyzed during the current study are not publicly available due to sensitive conservation status of the species of interest. Data may be available from the corresponding author on reasonable request.
